# On the Tensile Strength of Spark Plasma Sintered AlMgB_14_ Ceramics

**DOI:** 10.3390/nano12213805

**Published:** 2022-10-28

**Authors:** Pavel Nikitin, Ilya Zhukov, Dmitrii Tkachev, Alexander Kozulin, Alexander Vorozhtsov

**Affiliations:** Laboratory of Metallurgy Nanotechnologies, National Research Tomsk State University, Lenin Avenue 36, 634050 Tomsk, Russia

**Keywords:** AlMgB_14_, tensile strength, Brazilian test, spark plasma sintering, CASTEP

## Abstract

In this work, the structure, phase composition, hardness and tensile strength of the AlMgB_14_-based material obtained by spark plasma sintering (SPS) were investigated. According to the XRD results, the spark plasma sintered material contains 94 wt% AlMgB_14_ phase and 6 wt% spinel MgAl_2_O_4_. Analysis of the SEM images showed that the obtained AlMgB_14_ sample has a dense structure; the relative density of the sample is 98.6%. The average microhardness of the spark plasma sintered (SPSed) sample is 29 ± 0.88 GPa. According to the results of the Brazilian test, the tensile strength of AlMgB_14_ is 56 MPa. The fracture is characterized by a single straight tensile crack that divides the sample along the compression line into two halves. The type of fracture in the AlMgB_14_ sample can be characterized as a cleavage fracture due to crack growth occurring in accordance with the transcrystalline fracture. The tensile strength of the obtained material is in good agreement with the tensile strength of boride and oxide ceramics studied in other works.

## 1. Introduction

Superhard materials are characterized by a hardness of over 40 GPa [1]. Traditionally, diamond (HV = 80–100 GPa) and cubic boron nitride (HV = 60–80 GPa) are considered to be superhard materials [2]. It is known that solid materials have a high symmetry of the crystal lattice, the atoms of which have a strong interatomic bond. Thus, diamond has a strong sp^3^ covalent bond in the tetrahedral lattice configuration, while cubic BN has the cF8 diamond structure and belongs to the class of superhard materials due to the strong covalent bond of BN [2]. However, obtaining boron nitride in a cubic configuration is extremely expensive due to the need for high temperature and pressure in the synthesis process [1]. In this regard, the search for hard materials with excellent mechanical properties is an urgent scientific problem. To date, the attention of researchers is attracted by boron carbide (B_4_C), titanium diboride (TiB_2_) [3,4,5,6,7], cubic silicon carbide (β-SiC) [8], titanium carbide (TiC) [9,10], etc. Despite the fact that these materials cannot formally be classified as superhard, in the form of thin-film coatings, they demonstrate hardness values above 40 GPa [11].

It is important to note that all of the above materials have a simple symmetrical structure that satisfies the traditional paradigm of superhard materials [2,12,13]. However, since 2000, AlMgB_14_-based materials, which have a complex low-symmetric orthorhombic structure and, at the same time, high properties, have been actively studied. Aluminum magnesium boride (AlMgB_14_) has many excellent physical properties, such as high hardness (up to 32 GPa), relatively low density (2.59 g/cm^3^), oxidation resistance, excellent wear resistance and a very low coefficient of friction (up to 0.02) [2,14,15,16]. In combination with titanium diboride, materials based on AlMgB_14_ demonstrate hardness reaching 46 GPa [2], which classifies AlMgB_14_–TiB_2_ composite materials as superhard. As of yet, the main efforts of scientists have been directed at studying the influence of production methods (one-stage or two-stage sintering) and the composition of the initial mixture (ratio of initial elements, pre-reacted powders or mechanical mixture) on the structure, phase formation processes and properties of AlMgB_14_-based materials [13,14,15,16,17,18,19,20,21,22,23,24,25,26]. Today, it is traditionally considered that the best sintering methods for obtaining AlMgB_14_ are spark plasma sintering and hot pressing of AlMgB_14_ powder mixtures [2,14,15,16,17,21,22,23]. At the same time, the properties of the obtained materials are mainly influenced by the purity of the material (the presence of MgAl_2_O_4_ spinel in the samples leads to a significant decrease in the properties of AlMgB_14_ [27]). Nevertheless, despite the comprehensive number of publications devoted to the study of hardness, density, oxidation resistance, friction coefficients, coefficients of thermal expansion (CTE), and other properties of AlMgB_14_-based materials [2,14,15,16,17,18,19,28], there are no publications on their strength characteristics, in particular, on the tensile strength/flexural strength/compressive strength. At the same time, the tensile strength plays an important role in the analysis of the failure mechanism of structural ceramics and in the design of various structural ceramic parts; therefore, obtaining data on the strength characteristics of AlMgB_14_ is an important research task.

Tensile strength can be determined mainly by the direct tensile test and the Brazilian test. Conducting tensile experiments on ceramics is too difficult due to their low fracture strain. In turn, the Brazilian test, an indirect tensile method, is a popular choice for testing ceramic materials [29,30] due to the ease of material preparation and testing. Thus, the purpose of this work is to study the tensile strength using the Brazilian test, as well as the structure and the phase composition of AlMgB_14_ ceramics obtained by spark plasma sintering.

## 2. Materials and Methods

### 2.1. Process for Obtaining AlMgB_14_-Based Ceramics

AlMgB_14_-based ceramics were obtained by spark plasma sintering (DR. SINTER model SPS-625 Spark Plasma Sintering System, SPS SYNTEX INC. Ltd., Tokyo, Japan) of the Al_12_Mg_17_-B powder mixture at a pressure of 70 MPa and a temperature of 1400 °C. The working chamber was evacuated to a vacuum level of 6 Pa. Mechanical pressure was applied to the graphite die in the first minute of the sintering process and kept constant throughout the process. The sample was heated from room temperature to 1400 °C at a heating rate of 50 °C min^−1^. The diameter and thickness of the obtained samples were ~12.5 mm and 3 mm, respectively. The sintered sample was polished for further research. The characteristics of the raw powders are given in Table 1. To obtain a mixture of Al_12_Mg_17_-B, the powders of Al_12_Mg_17_ and amorphous black boron were mixed in an atomic ratio of 2:14 and mechanically activated (MA) in a planetary mill (Activator 4M, Engineering Plant “Activator”, Ltd., Dorogino, Russia) for 3 h in an argon atmosphere with a rotational frequency of 14 Hz. The mass ratio of grinding bodies to the powder mixture was 3:1. The average size of the resulting Al_12_Mg_17_-B powder mixture was 400 nm.

### 2.2. Brazilian Test

Tests at 24 °C and a loading rate of 0.75 mm/min were performed on an Instron 3369 double column testing machine. The specimens were placed in the testing machine between flat plates such that the loading force was applied along the center line of the specimen base. The ASTM D3967 (Brazilian test for brittle materials) recommended formula for calculating the tensile strength of a test material is given below:

*σ_t_* = 2*P*/*πLD* = 0.636*P*/*LD*

where *σ_t_* is the splitting stress (MPa), which is the value of the indirect tensile strength; *P*—the maximum applied force (N), is selected from the maximum value of the experimental curve “force–displacement”; *L* is the sample thickness (mm); *D* is the sample diameter (mm).

### 2.3. Characterization

X-ray diffraction analysis of the obtained sample was performed using a Shimadzu 6000 diffractometer with CuKα radiation and using the PDF-4 (Powder Diffraction File) database. The phase composition was refined using the Rietveld method. For this work, the CASTEP program code [31] was used to calculate the energies of the referenced and refined crystal lattices within the framework of the density functional theory (DFT). The exchange–correlation potential was treated within the generalized gradient approximation (GGA) using the Perdew–Burke–Ernzerhof (PBE-GGA) scheme [32]. A plane-wave cutoff energy of 500 eV was used. The microstructure of the obtained sample was determined using a QUANTA 3D microscope with energy dispersive spectroscopy (EDX). The density of the sintered sample was calculated using the Archimedes method. The Vickers hardness (HV) was determined using a Metolab-502 microhardness tester at a load of 1 kg (9.8 N). The loading time was 10 s. Ten indentations were made from different places of the sample.

## 3. Results

### 3.1. Microstructure and Phase Composition of the Obtained AlMgB_14_ Ceramics

**Figure 1:**
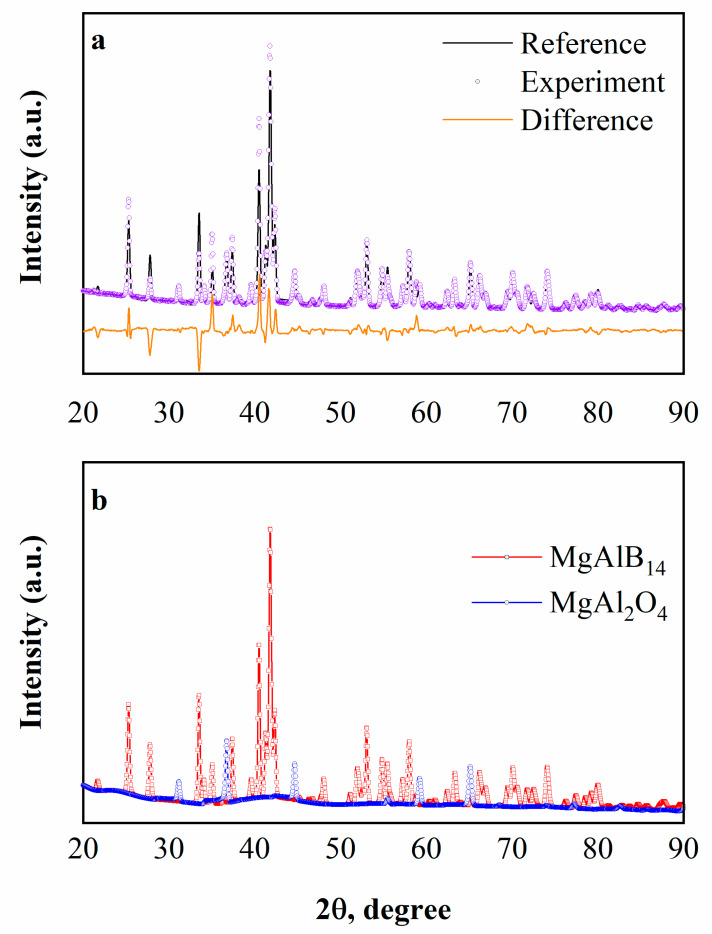
XRD patterns of the spark plasma sintered AlMgB_14_ samples: (**a**) 1—experimental XRD pattern (violet symbols), 2—integral intensity found using the Rietveld method (black line), 3—difference between experimental and integral intensities (orange line); (**b**) theoretical XRD patterns of the AlMgB_14_ and MgAl_2_O_4_ phases, found using the Rietveld method.

The results of the XRD analysis of the spark plasma sintered AlMgB_14_ sample are shown in Figure 1. Analysis of the contributions to the weight intensity of individual phases (Table 2, Figure 1) showed that in the obtained samples the main phases are AlMgB_14_ and MgAl_2_O_4_. The experimental XRD pattern (Figure 1, violet symbols) of the obtained composite is closely approximated by the calculated integral intensity (Figure 1, black line); the difference between them is insignificant (Figure 1, orange line).

Using the Rietveld method, the quantitative content of the phases was determined. It was found that the weight content of the AlMgB_14_ phase is dominant and amounts to 94 wt%, while the content of spinel MgAl_2_O_4_ in the obtained samples is no higher than 6 wt%. Structural parameters (a, b, c, α, β, γ) and the free energy (E) of the crystal lattices of AlMgB_14_ and MgAl_2_O_4_ in both the reference and the refined states are given in Table 2. As can be seen from Table 2, the structural parameters of the AlMgB_14_ lattice do not change. At the same time, the lattice volume V of the MgAl_2_O_4_ spinel changes significantly from 528.101 (reference state) to 520.688 (refined state) Å^3^. The lattice energy of MgAl_2_O_4_ in the refined state is higher than in the reference state, which indicates a lower lattice stability.

**Figure 2:**
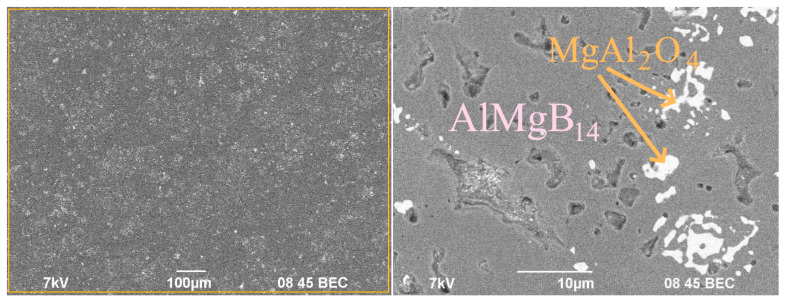
SEM images of the surface of the AlMgB_14_ sample.

The microstructure of the sintered sample is shown in Figure 2. As can be seen from Figure 2, spark plasma sintering of the Al_12_Mg_17_-B powder mixture leads to the formation of a dense homogeneous structure with inclusions of the oxide phase. According to the EDX results of the orange area in Figure 2, O, Al and Mg elements were found in the light areas (MgAl_2_O_4_ phase). In the dark areas, B, Al and Mg elements were found in the ratio corresponding to the AlMgB_14_ phase. EDX spectra are given in the Supplementary Materials. In the structure of the obtained sample, single pores with an average size of 3 μm were observed. The average grain size in the sintered samples is 3–5 µm. Based on the calculations using the Archimedes method, the relative density of the AlMgB_14_-based sample is 98.6% (if we take into account the 6% content of MgAl_2_O_4_ spinel, the theoretical density of the sample is 2.63 g/cm^3^).

### 3.2. Mechanical Properties of the Sintered Sample

The results of the hardness measurements of the obtained materials based on AlMgB_14_ are shown in Table 3. The average hardness of the obtained sample is 29 ± 0.88 GPa. The reported values of microhardness of the AlMgB_14_-based materials are 27.87 ± 0.97 [14], 26.7 ± 2.2 [15] and 26.1 [16] GPa, respectively. Thus, the value obtained in this work is in good agreement with the reported data [2,14,15,16,17,21,22].

**Figure 3:**
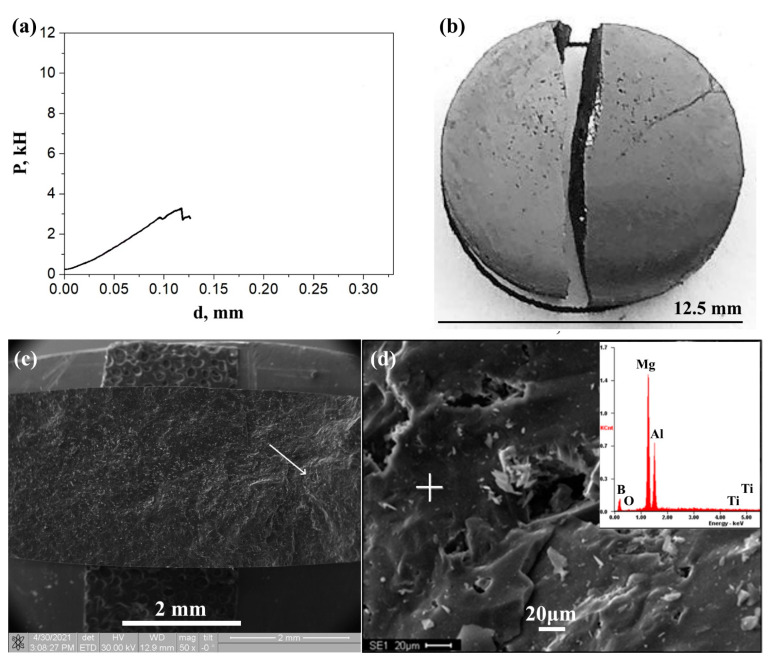
(**a**) The dependence of the change in the loading force on the AlMgB_14_ sample, obtained from the displacement during the experiment for the Brazilian test at a temperature of 24 °C and a loading speed of 0.75 mm/min; (**b**) the appearance of the AlMgB_14_ sample, fractured after the Brazilian test; (**c**) macro- and (**d**) micro-images of the fracture surface of the AlMgB_14_ sample.

Figure 3a shows the dependence of the change in the loading force applied to the AlMgB_14_ sample on the displacement during the experiment for the Brazilian test at a temperature of 24 °C and a loading rate of 0.75 mm/min (10^−3^ s^−1^). According to the obtained results, the tensile strength of AlMgB_14_ is 56 MPa.

## 4. Discussion

The appearance of the AlMgB_14_ sample, fractured after the Brazilian test, is shown in Figure 3b. Analyzing the image of a fractured AlMgB_14_ sample after the Brazilian test, we observe that the fracture is characterized by a single straight tensile crack that splits the specimen along the compression line into two halves. This failure is considered typical for loading conditions in the Brazilian test in terms of analytical strength theory assumptions [33,34,35]. At the same time, in [36,37], the fracture of ceramic materials after the Brazilian test was accompanied by a ternary fracture and multiple branching cracks. Such a fracture pattern can be explained by the inhomogeneous phase composition, which leads to dispersion strengthening due to inclusions with mechanical characteristics that differ from the base material. Thus, the AlMgB_14_-based material obtained in this study has a homogeneous phase composition, and therefore the fracture is characterized by a single straight crack.

The SEM image of the fracture surface of the AlMgB_14_ sample and the integrated elemental analysis of a typical microsection on the fracture surface are shown in Figure 3c,d. The crack initiation region is marked with a white arrow in Figure 3c. The initiation of a crack in the sample occurred not far from the central region of the sample, closer to the loading point. Most of the fracture surface, except for the crack initiation area, is relatively smooth without sharp drops and relief changes. Obviously, when under tension, the crack grew without resistance in local centers, which indicates the homogeneity of the composition and internal structure. The type of fracture can be characterized as a cleavage fracture due to crack growth occurring in accordance with the transcrystalline scenario. Multiple small fragments of the same elemental composition (Figure 3d) as the integral composition of the material were found on the fracture surface, with a predominance of Al, Mg and B chemical elements. The presence of other elements across the entire fracture surface is determined at the level of error (Table 4). A small number of micropores were also found, distributed evenly across the entire destruction surface with sizes not exceeding 20 μm.

The tensile strength of the obtained AlMgB_14_-based material is in good agreement with the tensile strength of the various ceramics studied in other works (Table 5). Apparently, the lower tensile strength of AlMgB_14_ compared to other ceramics can be associated with its complex low-symmetry orthorhombic crystal lattice and the presence of pores and microdefects in its structure. At the same time, as can be seen from Table 5, the composite material based on Al_2_O_3_–ZrO_2_ eutectic has the highest tensile strength. Due to the presence of the second phase in the structure of the material and due to phase transformations in the ZrO_2_ during destruction, the propagating cracks can branch and stop. In [2], a significant increase in the hardness of AlMgB_14_-based materials was reported due to the introduction of additives. With the addition of silicon, the hardness of AlMgB_14_ increased to 35 GPa; with the addition of titanium diboride, it increased to 46 GPa. In [38], the authors reported that AlMgB_14_ and TiB_2_ have an exceptionally strong bond due to very close values of their surface energies. In AlMgB_14_–TiB_2_ composite materials, AlMgB_14_ provides both good wetting and internal strength of the composite. In combination, these two borides can provide a high degree of mutual enhancement [38]. Based on the above, in the future, it will be of great interest to study the tensile strength of AlMgB_14_–TiB_2_ composite materials.

## 5. Conclusions

In this work, the AlMgB_14_-based ceramic material was obtained by spark plasma sintering. The mechanical properties, structure and phase composition were studied. The AlMgB_14_ phase content in the obtained sample was 94 wt% with a spinel MgAl_2_O_4_ content no higher than 6 wt%. According to the results of density and hardness measurements, the relative density of the sintered AlMgB_14_-based material was 98.6% with an average microhardness of 29 GPa. According to the results of the Brazilian test, the tensile strength of AlMgB_14_ was 56 MPa. The fracture is characterized by a single straight tensile crack that divides the sample along the compression line into two halves. The type of fracture of the AlMgB_14_ sample can be characterized as a cleavage fracture due to its crack growth appearing in accordance with the transcrystalline fracture. The tensile strength of the obtained material is in good agreement with the tensile strength of boride and oxide ceramics studied in other works.

## Figures and Tables

**Table 1:** Characteristics of the raw powders.

Powder	Average Particle Size	Purity, %
Al_12_Mg_17_	15 μm	≥99.2
Amorphous B	600 nm	≥98.7
MA-Al_12_Mg_17_-B	400 nm	≥98.8

**Table 2:** Structural parameters of lattices.

Phase	State	a, Å	b, Å	c, Å	α = β = γ	V, Å^3^	E, eV
AlMgB_14_	Reference	5.850	8.111	10.310	90	489.202	−8451.883
	Refined	5.851	8.112	10.311	90	489.394	−8451.884
MgAl_2_O_4_	Reference	8.083	8.083	8.083	90	528.101	−22767.860
	Refined	8.045	8.045	8.045	90	520.688	−22767.475

**Table 3:** The results of measuring the hardness of the obtained sample.

Measurement Number/HV										HV_av_, GPa
1	2	3	4	5	6	7	8	9	10	29.00 ± 0.88
2743.9	2795.6	2858.0	3050.8	2903.4	2805.4	2795.6	2931.3	3073.3	3073.3	

**Table 4:** Results of the elemental analysis of a typical microsection on the fracture surface.

Element	B	Al	Mg	O	Ti
Wt%	87.6	4.10	8.00	0.25	0.05

**Table 5:** Comparison of the tensile strength of AlMgB_14_ with other ceramics.

Material	σ_t_, MPa	Reference
AlMgB_14_	56.0	This work
Al_2_O_3_–ZrO_2_(Y_2_O_3_) eutectics	80.0	[39]
HfB_2_	53.8	[40]
TiB_2_	60.2	[40]
ZrB_2_	53.3	[40]

## Data Availability

The data presented in this study are available in the article.

## Supplementary Materials

The following supporting information can be downloaded at: https://www.mdpi.com/article/10.3390/nano12213805/s1. EDX spectra are given in the Supplementary Materials.
