# Supplementary figures and images for: On the Tensile Strength of Spark Plasma Sintered AlMgB14 Ceramics
Source: Nanomaterials (Basel). 2022 Oct 28;12(21):3805. doi: 10.3390/nano12213805 (PMC9659233; doi:10.3390/nano12213805)

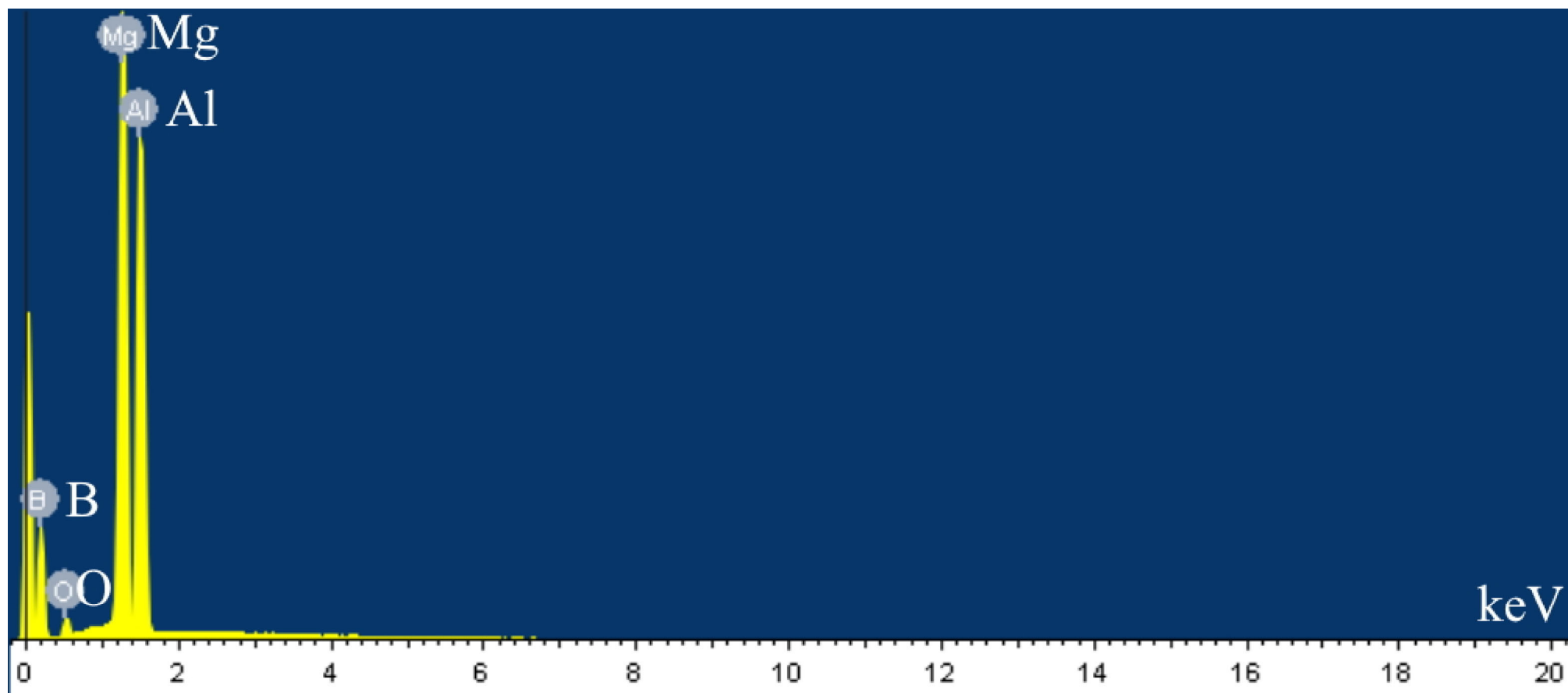

Supplement: Supplementary file 1 [file nanomaterials-12-03805-s001.zip › nanomaterials-1987718-supplementary.pdf]
